# Multimodal Brain-Tumor Segmentation Based on Dirichlet Process Mixture Model with Anisotropic Diffusion and Markov Random Field Prior

**DOI:** 10.1155/2014/717206

**Published:** 2014-09-01

**Authors:** Yisu Lu, Jun Jiang, Wei Yang, Qianjin Feng, Wufan Chen

**Affiliations:** ^1^Electronic Engineering Department, South China Institute of Software Engineering, Guangzhou 510990, China; ^2^Key Lab for Medical Image Processing, Southern Medical University, TongHe, Guangzhou 510515, China

## Abstract

Brain-tumor segmentation is an important clinical requirement for brain-tumor diagnosis and radiotherapy planning. It is well-known that the number of clusters is one of the most important parameters for automatic segmentation. However, it is difficult to define owing to the high diversity in appearance of tumor tissue among different patients and the ambiguous boundaries of lesions. In this study, a nonparametric mixture of Dirichlet process (MDP) model is applied to segment the tumor images, and the MDP segmentation can be performed without the initialization of the number of clusters. Because the classical MDP segmentation cannot be applied for real-time diagnosis, a new nonparametric segmentation algorithm combined with anisotropic diffusion and a Markov random field (MRF) smooth constraint is proposed in this study. Besides the segmentation of single modal brain-tumor images, we developed the algorithm to segment multimodal brain-tumor images by the magnetic resonance (MR) multimodal features and obtain the active tumor and edema in the same time. The proposed algorithm is evaluated using 32 multimodal MR glioma image sequences, and the segmentation results are compared with other approaches. The accuracy and computation time of our algorithm demonstrates very impressive performance and has a great potential for practical real-time clinical use.

## 1. Introduction

Magnetic resonance (MR) imaging technology has been widely applied to medical diagnosis systems, and the accuracy of many diagnosis systems is mainly based on the quality of the images acquired. However, the images obtained by magnetic resonance imaging usually contain heavy noise and the effects of the biasing field, which will degrade the quality of the images and make the subsequent postprocessing of the images, such as segmentation, classification, and detection, difficult. The noise and biasing-field effects in MR images sometimes even affect the evaluation of human segmentation. Hence, segmentation for MR images automatically becomes a very challenging task.

In general, the essential segmentation task is to label the different parts of the object image. Thus, the class number of the parts for the segmented image is a very important parameter. As is well-known, the number of clusters for medical image segmentation is difficult to initialize as a constant before clustering. Therefore, most classical segmentation methods for medical images [1, 2] specify the number of clusters before clustering as a matter of clinical experience, even some advanced methods estimate the number of clusters from educated guesses or prior knowledge [1, 2].

Thus, an automatic segmentation method without initialization of the number of clusters is very attractive for medical images, which can greatly improve the effectiveness and efficiency for clinical diagnosis. In this paper, we present an algorithm that utilizes MDP models [3, 4], which can automatically segment a medical image without initializing the number of clusters before clustering. In the MDP method, the number of clusters is not a constant number but is instead defined as a level of cluster resolution by the control parameter.

The classical MDP model was proposed by Antoniak [3] and Ferguson [4], which has been used in the fields of statistical problems and language processing [5, 6]. In this study, we incorporate this model into medical brain-tumor image segmentations. Because the computing speed and convergence of the classical MDP method is not very good for clinical image clustering, we introduce anisotropic diffusion and Markov random fields (MRF), which are combined with the classical MDP models to construct our algorithm. We apply this algorithm to segment active tumor tissue and edema in MR brain-tumor images. The experimental results show that the accuracy and convergence of the proposed algorithm are extraordinary. In particular, the computing time is significantly lower than the classical MDP algorithm.

## 2. Methods

### 2.1. Anisotropic Diffusion Filter

In order to alleviate the effects of noise, noise reduction is generally used to remove or reduce the noise before segmentation, classification, and detection. Noise reduction is an important image processing method that has wide application in various fields [7, 8]. The key to noise reduction is to reduce the noise without deteriorating the important features in the images. Thus, noise reduction has two goals. One is to remove the noise from the images, and the other is to preserve the important features such as the edges in the images.

Depending on the noise type, a noisy image can be generally modeled as one of the two models: a linear model and a nonlinear model. If the noise is independent of the image, then it can be described by a linear model (an additive noise model). An MR image is generally modeled as an additive noise model. However, traditional noise reduction technologies such as Gaussian and median filters are not very effective in reducing the noise because the distribution of the noise is not Gaussian; it is assumed to be a Rician distribution.

Here, we utilize an anisotropic diffusion (AD) filter [9] for noise reduction in MR images. AD is a nonlinear filtering method, which encourages diffusion in the homogeneous region while inhibiting diffusion at the edges. The partial differential equation (PDE) of anisotropic diffusion is as follows in the continuous domain:

(1) ∂I∂t=div⁡c∇I∇I,It=0=I0,

where ∇ is the gradient operator, div⁡ is the divergence operator, *I* is the initial image, and *c*(·) is the diffusion coefficient, which is often chosen such that *c*(*x*) → 0 as *x* → *∞*. Further, *c*(·) should be monotonically decreasing so that diffusion decreases as the gradient strength increases, and the diffusion stops across edges. Several expressions for *c*(·) have been suggested for diffusion [10]:

(2) cx=exp⁡⁡−xq2,cx=11+x2−q2/k21+q2,

where *q* is a parameter to control the extent of diffusion. In our paper, a discrete form of (1) is given by

(3) $$\begin{array}{c} I_{s}^{t+\Delta t}=I_{s}^{t}+\frac{\Delta t}{\bar{\eta_{s}}}\underset{p\in \bar{\eta_{s}}}{\sum }c\left(\nabla I_{s,p}^{t}\right)\nabla I_{s,p}^{t}, \end{array}$$

where *I*_*s*_^*t*^ is the discretely sampled image, *s* denotes the pixel position in a discrete 2D grid, ∇*t* is the time step size, $\bar{\eta_{s}}$ represents the spatial neighborhood of pixel *s*, $\left|\bar{\eta_{s}}\right|$ is the number of pixels in the window, and

(4) $$\begin{array}{c} \nabla I_{s,p}^{t}=I_{p}^{t}-I_{s}^{t},\;\forall p\in \bar{\eta_{s}}. \end{array}$$

### 2.2. Dirichlet Process Mixture Models

We consider the Dirichlet process mixture (MDP) model as the statistical model in our study. The MDP model is a kind of nonparametric Bayesian model. A MDP model comprises three principal components: A parametric likelihood function *F*, a probability distribution *G*_0_, which is referred to as the base measure, and a Dirichlet process DP(*αG*_0_), which is parameterized by the base measure and a positive constant *α* ∈ *R*+. In our paper, the MDP model is a set of distinct classes, which is assumed to be generated by the observed data *x*_1_,…, *x*_*n*_. Every class (indexed by *k*) has a generative distribution, which is described by the likelihood *F*, and is characterized by the parameter value *θ*_k_*. Therefore, the data within the cluster is generated according to *x* ~ *F*(·∣*θ*_k_*). In contrast to the parametric model, the number of classes is not a constant, which will change during the sampling process.

Data generation of *n* data values *x*_1_,…, *x*_*n*_ according to the MDP model can be summarized as

(5) x1,x2,…,xn~F· ∣ θi∗,θi~Pθi,P~DPαG0.

Therefore, the Dirichlet process can be considered as a distribution of distributions and assumed as the prior distribution in the Bayesian setting. These principals can be used to determine model selection and clustering problems during machine learning.

For this reason, the MDP models can be thought of as mixture models. The number of mixture components in the mixture model is a random variable, which can be evaluated from the input dataset. The term clustering method is used to describe an unsupervised learning measurement, which groups a set *x*_1_, *x*_2_,…, *x*_*n*_ of input data into an independent class.

If the given set of samples *θ*_1_,…, *θ*_*N*_*C*__ are already drawn from the random measure *G*, we can integrate the measure and obtain conditional prior distribution of a new sample *θ*_*n*+1_:

(6) pθn+1 ∣ θ1,…,θn=1n+α∑i=1nδθiθn+1+αn+αG0θn+1.

The number of clusters is denoted by *N*_*C*_. Each of these classes is represented by its associated parameter value, denoted *θ*_k_*, for class *k* ∈ {1,…, *N*_*C*_}. The combination with a parametric likelihood *F*, as in (5), results in a single, fixed likelihood function *F*(·∣*θ*_k_*) for each class *k*. Hence, the model is a mixture model with *N*_*C*_ parametric components *F*(·∣*θ*_k_*) and a base measure term (“zero” component), which is responsible for creating the new classes.

In the classical parametric mixture model applied to a clustering problem, the number of clusters is determined by the fixed number of parameters. If the number of clusters is changed, the structure of the parametric mixture model should be changed accordingly. However, the nonparametric MDP models provide a new description of clustering methods that could adjust the number of clusters without changing the models. Thus, this property is the required precondition for Bayesian inference of the number of clusters, which defines *N*_*C*_ as a random variable in the model framework, rather than a constant.

### 2.3. Cost Function Constrained with the MRF

This work combines nonparametric mixture of Dirichlet process models with MRF models to enforce spatial constraints. Many computer vision problems involve the MRF model [9]. In our study, we obtain a model capable of combining the clustering and model selection performed by the MDP with the smoothness constraints on the class labels, which is reasonable to assume a spatially coherent class structure, for the MRF constraint encourages adjacent points in the image to be assigned into the same class.

The vectorial input data *x*_1_,…, *x*_*n*_ is considered in the clustering problem. Every point *x*_*i*_ is assumed to be generated with the parameter vector *θ*_*i*_. Two points are assigned into the same cluster if their respective parameter vectors are identical.

To combine the MDP model with a MRF, we restrict the choice of MRF constraints to pairwise difference priors [10], in which the MRF is commonly used to model spatial smoothness of label field. The MRF definition is based on undirected neighborhood graph *N*.

The MRF prior Π comprises two components:

(7) $$\begin{array}{c} \prod \left(\theta \right)\propto P\left(\theta \right)M\left(\theta \right). \end{array}$$

*P* is a parametric prior on the parametric *θ*, which will be referred to as the initial prior. In our paper, the initial prior *P* is drawn from the DP model (described in Section 2.2). *M* is a MRF contribution term, which can be defined as

(8) $$\begin{array}{c} M\left(\theta_{i}\right)\propto \exp\left(-H\left(\theta_{1},\ldots ,\theta_{n}\right)\right), \end{array}$$

where *H* is the cost function defined on neighborhood graph *N*. The term *M* is used as the model smoothness constrains, which is conditional on the neighborhood of feature.

Therefore, the resulting generative model is summarized by

(9) x1,x2,…,xn~Fxi ∣ θi∗,θi~Mθi ∣ θ−iPθi,P~DPαG0.

In our paper, MRF cost function with the parameters *θ*_*i*_ is defined as

(10) $$\begin{array}{c} H\left(\theta_{i}\;|\;\theta_{-i}\right)≔\underset{l\in \partial \left(i\right)}{\sum }{||\theta_{i}-\theta_{l}||}^{2}. \end{array}$$

We write *l* ∈ ∂(*i*) to denote that the feature of index *l* is the neighbor of feature *i*. The distribution of resulting conditional prior distribution *M*(*θ*_*i*_∣*θ*_−*i*_) ∝ ∏_*l*∈∂(*i*)_exp⁡⁡(−||*θ*_*i*_−*θ*_*l*_||^2^) will have similar parameter values at the neighbor sites.

Most clustering problems do not define an order for the class labels. In our paper, two class labels are either identical or different. Hence, the cost function is expressed with the binary concept of similarity as

(11) $$\begin{array}{c} H\left(\theta_{i}\;|\;\theta_{-i}\right)=-\lambda \underset{l\in \partial \left(i\right)}{\sum }\omega_{il}\delta_{S_{i},S_{l}}, \end{array}$$

where *δ* is the Kronecker symbol and *λ* is the constraint parameter, which is a positive constant. *ω*_*il*_ are the edge weights. The class indicators *S*_*i*_ and *S*_*l*_ specify the different classes of all neighbors by the parameters *θ*_*i*_ and *θ*_*l*_. Therefore, if *θ*_*i*_ defines a class different from the classes of all neighbors, exp⁡(−*H*) = 1, whereas exp⁡(−*H*) will increase if at least one neighbor is assigned to the same class.

Such a cost function can be used to express the smoothness constraints on the cluster labels because they promote the smooth assignment of the adjoining sites. Further, the results of the segmentation algorithm can be smoothed with the label constraints.

**Figure 1:**
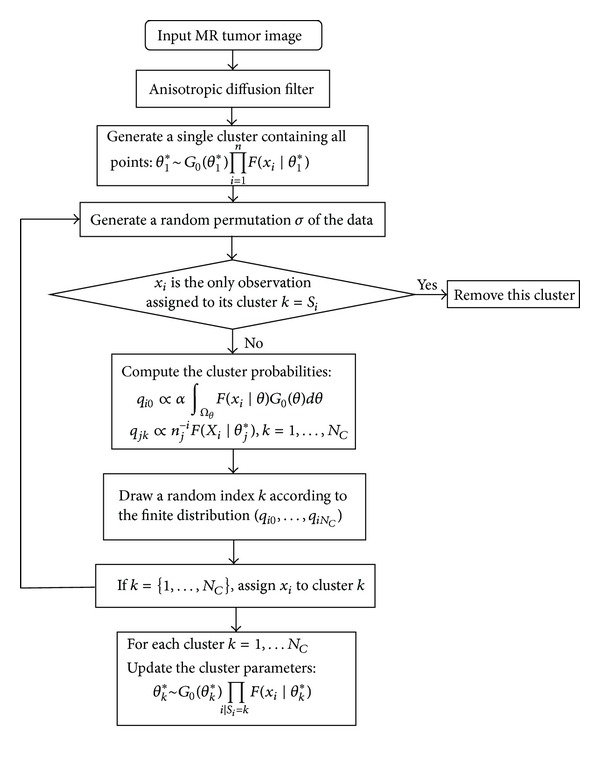
Flow chart of our algorithm.

In summary, we can obtain the algorithm and the complete flow chart as shown in Figure 1.

### 2.4. Data Acquisition

The brain-tumor image data used in this work were obtained from the MICCAI 2012 Challenge on Multimodal Brain Tumor Segmentation (http://www.imm.dtu.dk/projects/BRATS2012) organized by B. Menze, A. Jakab, S. Bauer, M. Reyes, M. Prastawa, and K. Van Leemput. This database contains fully anonymized images from the following institutions: ETH Zurich, University of Bern, University of Debrecen, and University of Utah.

The brain-tumor image data contain information on 120 subjects with gliomas; 55 images are real patient data, and 65 images are synthetic data. A total of 80 images out of 120 images with ground truth data are treated as training data (Table 1). The tumor and edema regions are manually delineated by the experts in the clinical images and synthetic data, which is used as the segmentation criterion and called “ground truth” (GT) in this paper. The following accuracy analysis is used to compare our segmentation results to the GT.

**Figure 2:**
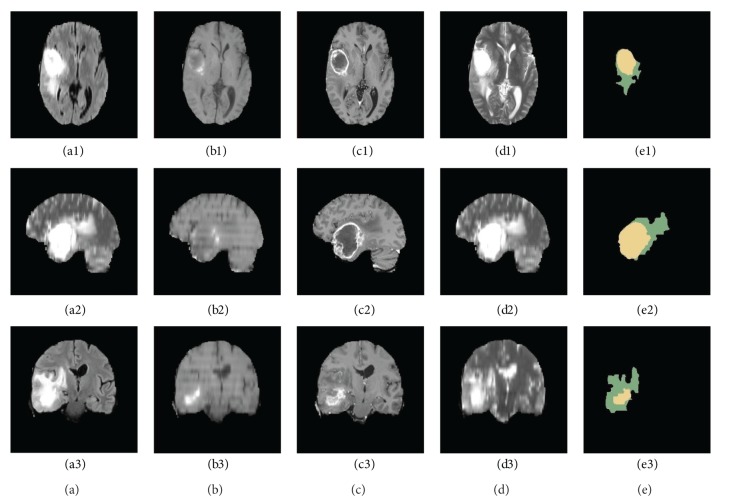
Three-dimensional multimodal slices of clinical high-grade glioma MR images.

For each patient, T1, T2, FLAIR, and contrast-enhanced T1-weighted (T1C) MR images were available. All volumes were linearly coregistered to the T1 contrast image, skull stripped, and interpolated to 1 mm isotropic resolution. Some slices of a high-grade glioma patient are shown in Figure 2. 3D MRI slices of a high-grade glioma patient are shown in the three rows of Figure 2, such as the transverse section (images a1 to d1), median sagittal section (images a2 to d2), and coronal section (images a3 to d3). The first four columns show the FLAIR (images a1 to a3), T1 (images b1 to b3), T1C (images c1 to c3), and T2 (images d1 to d3) MR images, and the last column (images e1 to e3) shows the GT of the 3D FLAIR high-grade clinical glioma MR images segmented by the experts, in which the yellow region marks the tumor core, and the green region marks the edema.

We applied our algorithm to 32 patients among the MICCAI2012 data sets, the data among which there are 10 clinical high-grade glioma patients, 6 clinical low-grade glioma patients, 10 synthetic high-grade glioma patients, and 6 synthetic low-grade glioma patients.

### 2.5. Multimodal Tumor Image Segmentation

In most studies, segmentation is conducted for single modal brain-tumor MR images, which targets the gross tumor volume (GTV) of the brain tumors in T1C MR images. In this study, we introduce a new algorithm to segment multimodal MRI images (T1C and FLAIR), which include edema segmentation and evaluate multimodal MR tumor images. Our algorithm is applied to FLAIR and T1C volumes to segment the clinical tumor volume (CTV = GTV + edema).

From the descriptions by the medical experts in the literature [11, 12], the different modalities of the images enhance the unique information in the same region of the human body. The MR signals of the hydrogen atom are restrained in the FLAIR images, and the contrast of the cerebrospinal fluid is lower than the surrounding tissues. In addition, the highlighted abnormal “enhancement” regions indicate the tumor core region in the T1C images. This medical knowledge encourages us to utilize the image difference feature between the two modalities. Our segmentation results are integrated by setting the T1C segmentation results as the tumor labels and the difference area of the FLAIR segmentation minus the T1C segmentation as the edema labels:

(12) $$\begin{array}{c} V_{\text{Edema}}=\left\{x\in V_{\text{FLAIR}}\;|\;x\notin V_{\text{T}1\text{C}}\right\}. \end{array}$$

**Figure 3:**
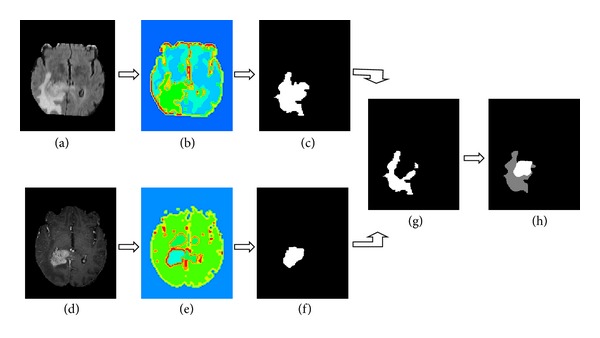
Segmentation of a multimodal tumor image by the our algorithm: (a) FLAIR high-grade glioma MR slice, (b) segmentation results of (a) by our algorithm, (c) entire tumor mask, (d) TIC high-grade glioma MR slice, (e) segmentation results of (d) by our algorithm, (f) active tumor mask, (g) edema mask, and (h) entire tumor mask; the gray region indicates the edema, and the white region indicates the tumor core.

The execution process is depicted in Figure 3.

## 3. Experimental Results and Discussion

### 3.1. Constraint Parameter of MDP/MRF

The experiments presented in this section were conducted on single model brain-tumor images and multimodel brain-tumor images. Besides the visual quality of the segmentation results, we especially study the model selection question: how does the constraint parameter of the MDP/MRF algorithm influence the model selection?

**Figure 4:**
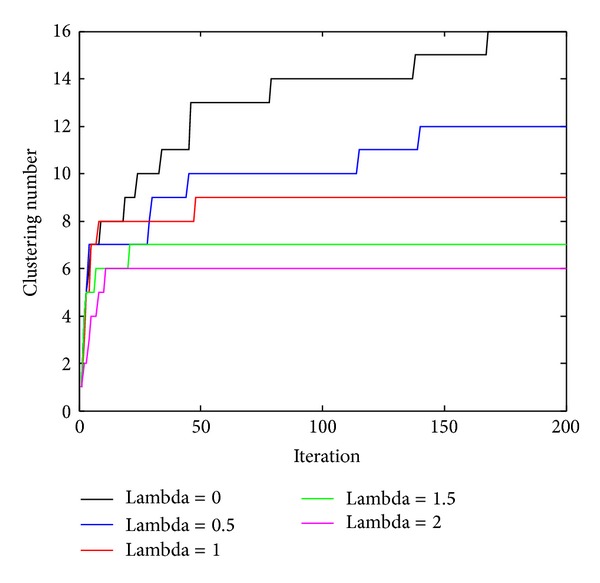
Clustering assignments with different constraint parameter *λ*.

A clinical high-grade glioma T1C MR image is segmented by the MDP/MRF algorithm. Our method repeatedly computes clustering solutions on randomly chosen subsets of the input data and evaluates the predictive power of the obtained cluster model on the remaining data. The burn-in phase of the Gibbs sampling algorithm is assumed to have terminated once the number of assignments changed per iteration remains stable below 1% of the total number of sites. This condition is met after at most 200 iterations. The behavior of the clustering assignments during the sampling process is visualized by the plot in Figure 4. In all the cases, the algorithm is executed with different constraint parameter *λ* and takes about 200 iterations to stabilize (the curves become constant apart from fluctuations). The splitting behavior of the algorithm differs significantly among all the cases: in the unconstrained case, lager batches of sites are reassigned at once to new clusters (visible as jumps in the diagram). Assignments change gradually by the increasing of constraint parameter *λ*, which means more adjoining sites are assigned into the same cluster.

### 3.2. Single Modal Tumor Image Segmentation

#### 3.2.1. Design of Experiments

**Figure 5:**
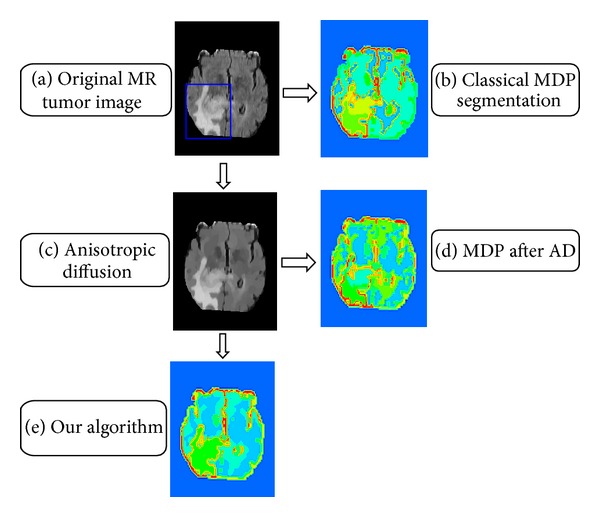
Segmentation of a single modal tumor image.

The operation of our algorithm, which shows the main steps and the corresponding segmentation results, is shown in Figure 5 compared with classical MDP model segmentation. A clinical high-grade glioma T1C MR image is segmented in Figure 5. Figure 5(a) shows the clinical MR image, and the focus of the tumor is indicated by a blue rectangle. Figure 5(b) shows the segmentation of the MR brain-tumor image by directly using the classical MDP segmentation algorithm, in which the number of clusters is 11. The clinical brain-tumor image is anisotropically diffused in Figure 5(c). After the original MR image is anisotropically diffused, the classical MDP algorithm is used to segment the image in Figure 5(d), in which the number of clusters is eight. In Figure 5(e), segmentation is carried out by our algorithm, and the number of clusters is five.

It is important that the number of clusters is decreased from the MDP results to the algorithms. Because the number of clusters is very large for the classical MDP algorithm, the tumor region is divided into many fragments, and it is difficult to accurately segment the entire tumor. This is because more accurate segmentation boundaries of the tumor region are created by our algorithm than the classical MDP. Hence, it is more appropriate to use our algorithm to segment tumor images.

#### 3.2.2. Convergence Analysis

**Figure 6:**
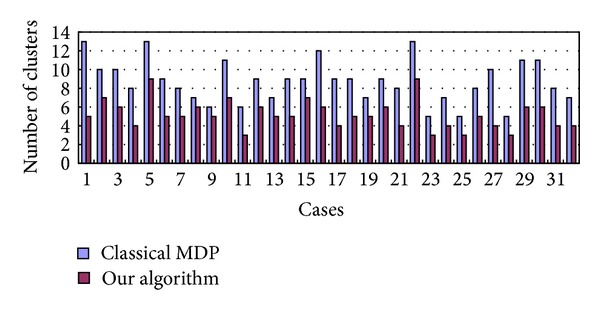
Comparative analysis for the number of clusters between classical MDP (blue bar) and our algorithm (red bar).

The clustering class numbers for the 32 active tumor segmentation results are shown in Figure 6, which were segmented by the MDP algorithm and our algorithm. There are 200 iterations in both algorithms. The numbers of clusters are listed in the table in Figure 6. The results indicate that all the class number results obtained by the current algorithm are less than those of the classical MDP algorithm. Thus, the modified model could properly control the segment class number in order to reduce the convergence time.

**Figure 7:**
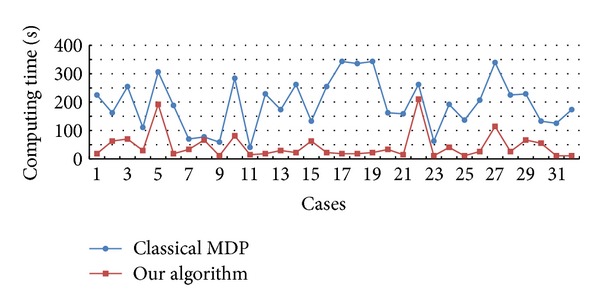
Comparative analysis for the convergence time of MDP (blue) and our algorithm (red).

The results in Figure 7 show the computation time of 32 brain-tumor MR images segmented by the MDP algorithm and the algorithm. The computing time is related to the size of the tumor region. The experiment results indicate that the average convergence time of the algorithm is 42.04 s, which is much shorter than that of the classical MDP algorithm (218.37 s). The results demonstrate that the convergence speed of the segmentation results of the algorithm is significantly faster than the classical MDP algorithm.

Because of the high computational complexity of the classical MDP algorithm without considering the region/boundary features, the application of the classical MDP algorithm for complex target segmentation is limited. The AD filter and MRF smooth prior are introduced into the MDP model, and the target segmentation can be obtained with fewer iterations by our algorithm (Figure 7). Therefore, the computing time for convergence of our algorithm is much shorter than the classical one (Figure 7). All the results show that the algorithm is more efficient and robust.

### 3.3. Multimodal Tumor Image Segmentation Results

#### 3.3.1. Segmentation Results

**Figure 8:**
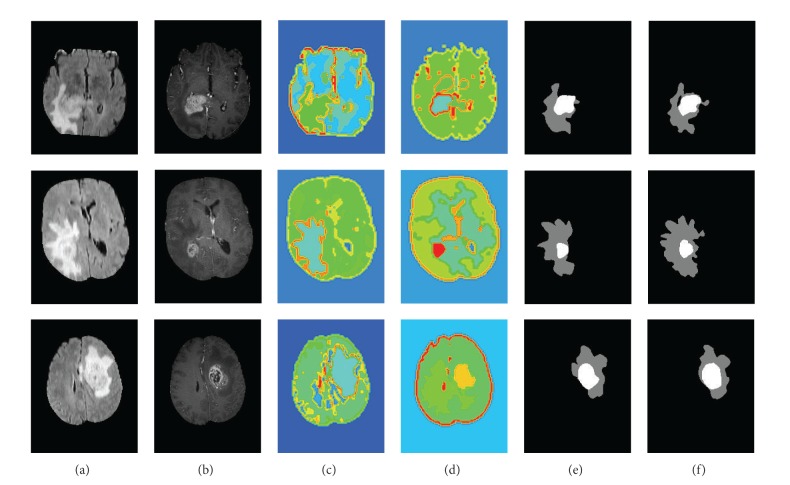
Segmentation results of the active tumor and edema in the real clinical glioma MR images.

**Figure 9:**
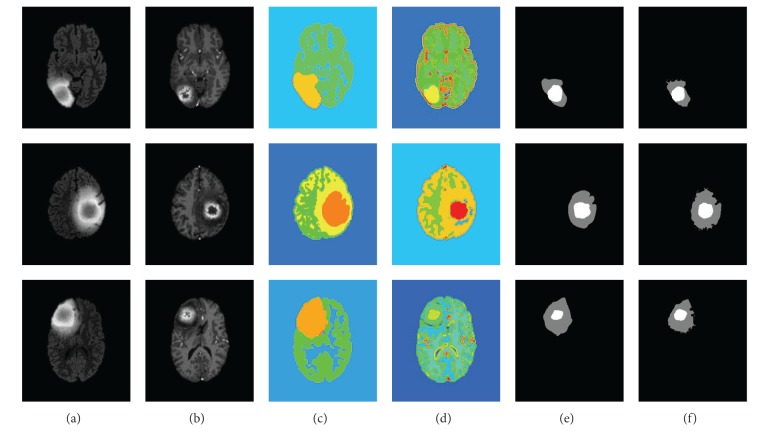
Segmentation results of the active tumor and edema in the synthetic glioma MR images.

Some segmentation results of multimodal tumor images are presented in Figures 8 and 9.  Figure 8 shows the real clinical data segmentation results, and Figure 9 shows the results of the synthetic data. The original FLARE and TIC MR images are shown in the line (a) and line (b). The line (c) shows segmentation of the FLARE MR images, which show the segmentation results of the entire tumor region. In addition, the T1C MR images are segmented, and the tumor core segmentation results are shown in the line (d). The last two rows show the GT masks (line (e)) and the segmentation masks by the algorithm (line (f)), where the white region indicates the tumor core mask, and gray region indicates the edema mask. The results in Figure 8 and Figure 9 show great similarity between the GT and our segmentation results.

Comparatively, the tumor boundaries of the real patient data (Figure 8(f)) were more blurry than those of the synthetic data (Figure 9(f)). Therefore, the tumor segmentation performance was better in the synthetic data than that in the real patient data. The edema boundaries of both real patient data and synthetic data were quite blurry, which led to more inaccurate segmentation performance in the edema regions than those in the tumor regions.

#### 3.3.2. Comparison with Other Methods

**Figure 10:**
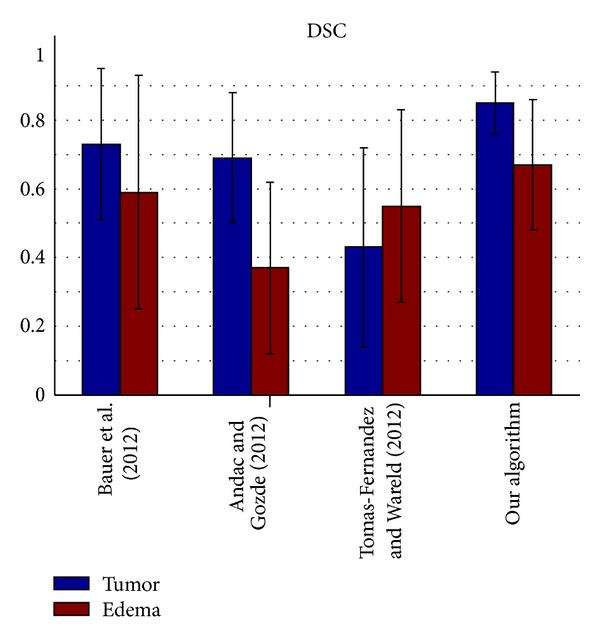
Dice similarity coefficient evaluation of the active tumor and edema segmentation results.

**Figure 11:**
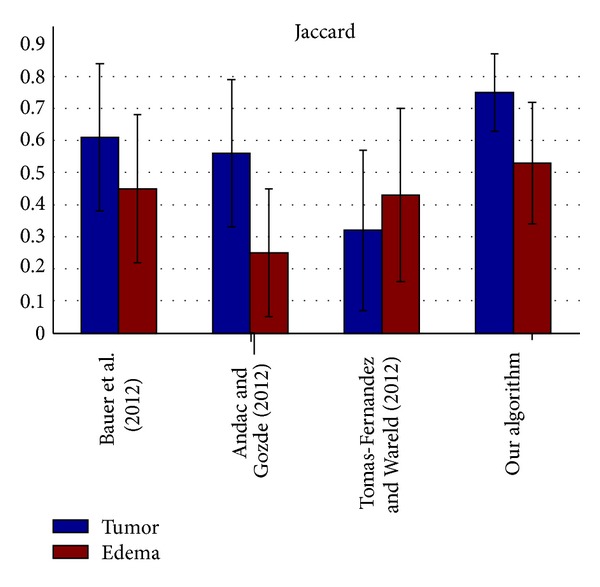
Jaccard similarity coefficient evaluation of the active tumor and edema segmentation results.

**Figure 12:**
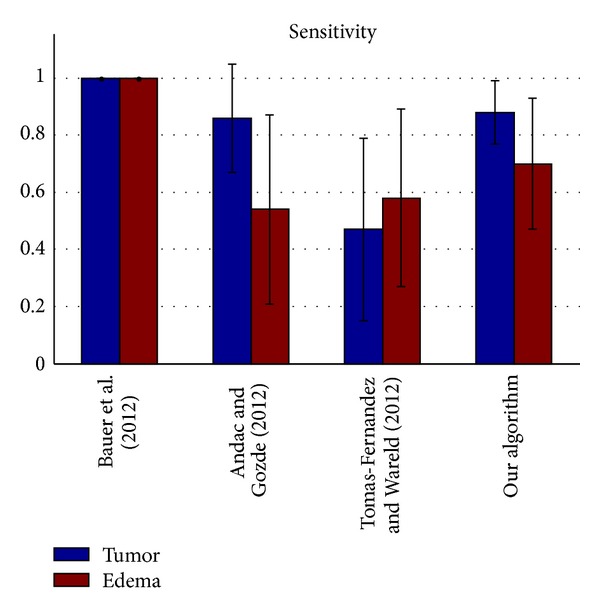
Sensitivity evaluation of the active tumor and edema segmentation results.

**Figure 13:**
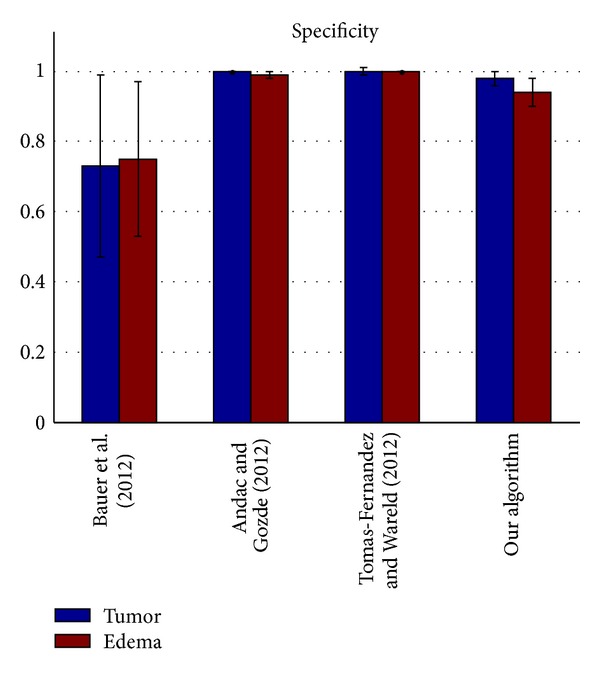
Specificity evaluation of the active tumor and edema segmentation results.

The segmentation results of the testing data were evaluated by the online evaluation tool in the evaluation platform. This evaluation platform contains an archive of all uploaded results, which enables segmentation methods to be objectively benchmarked and compared with each other. The tumor region in the patient data was subdivided into two classes: active tumor core and edema. Figures 10, 11, 12, and 13 show the tumor segmentation results with different methods evaluated by the online evaluation tool.

In order to obtain a better understanding of the data and the performance of our algorithm, we perform four further measurements as a statistical analysis of the segmentation accuracy. For each segmentation result, the true positive (TP), true negative (TN), false positive (FP), and false negative (FN) pixels are counted. The four measurements are defined as follows [13, 14]:

(13) TP=R∩T,TN=R∪T¯,FP=R∩T¯,FN=R¯∩T,

where *T* is the true set and *R* is the result set.

For all algorithms, the following four statistical analysis accuracy metrics are computed: Dice similarity coefficient (DSC), Jaccard similarity (Jaccard), Sensitivity (Sens.), and Specificity (Spec.) [13, 14].

 Dice coefficient is as follows:
  (14) $$\begin{array}{c} \text{DSC}=\frac{2*\text{TP}}{\left(\text{FP}+\text{TP}\right)+\left(\text{TP}+\text{FN}\right)}. \end{array}$$
 Jaccard similarity is as follows:
  (15) $$\begin{array}{c} \text{Jaccard}=\frac{\text{TP}}{\text{FP}+\text{TP}+\text{FN}}. \end{array}$$
 Sensitivity (fraction of positives that are correctly detected) is as follows:
  (16) $$\begin{array}{c} \text{Sens}.=\frac{\text{TP}}{\text{TP}+\text{FN}}. \end{array}$$
 Specificity (fraction of negatives that are correctly detected) is as follows:
  (17) $$\begin{array}{c} \text{Spec.}=\frac{\text{TN}}{\text{TN}+\text{FP}}. \end{array}$$

The top four results of statistical analysis are listed from Figure 10 to Figure 13 (Bauer et al. [15], Andac and Gozde [16], and Tomas-Fernandez and Wareld [17] and our method). At the time of writing of this study, the proposed method was in the second place about Sensitivity and in the third place about Specificity. Moreover, the proposed method is in the first places about Dice similarity coefficient and Jaccard similarity, respectively. These results indicate that the proposed method is comparable to other state-of-the-art methods.

Theoretically, the other three approaches adopted pixel intensity rather than sufficient features from the region and boundary term construction, and the clustering number of the other approaches should be initialized by human intervention. Therefore, the other algorithms exhibit lower performance owing to segmentation leakage in some cases, making the segmentation prone to failure. Moreover, our algorithm exhibits higher sensitivity and higher specificity. On the other hand, the algorithm exhibits a relatively higher mean value but a lower coefficient of standard variation (mean/std) on average compared with the other algorithms used for validation. This means our algorithm can predict a lesion in an MR image sensitively and precisely, making little misjudgments.

## 4. Conclusions 

Our algorithm combined the AD filter and the MRF constraint. Therefore, the AD filter can help to remove the noise from the images and preserve important features such as edges in the images. In addition, the MRF smooth prior constraint is set as the region constraint prior of the cost function to help promote the smooth assignment of the adjoining sites. All of the improvements to the classical MDP algorithm lead to the modified nonparametric algorithm that can effectively be used in clinical tumor segmentation.

To summarize, we have developed a new nonparametric image segmentation algorithm for MR multimodal brain-tumor images. On the basis of actual clinical and synthetic data, our algorithm demonstrates very impressive performance. By taking advantage of the features of multimodal MR images, such as T1C and FLAIR modal features, our algorithm could segment active tumors and edemas, respectively, from the MR tumor images correctly. Furthermore, it significantly reduces the computation time. Hence, this algorithm can be used for complicated brain-tumor images with high noise and the biasing-field effect in real-time clinical use.

However, there are some limitations in our approach. In the proposed algorithm only two types of MR modal features, T1C and FLAIR modal features, are used. The other types of MR modal features, such as T1 and T2, are not utilized. Therefore, our algorithm used in glioma MR image segmentation cannot separate the other types of tumors in the brain lesion images. In the further work, we also plan to extend the algorithm into 3D and realize a 3D tumor reconstruction system, which can be better used in clinical applications.

## Figures and Tables

**Table 1:** Cases of glioma patients in the testing data set.

High-grade glioma patients	Clinical data	20
	Synthetic data	25

Low-grade glioma patients	Clinical data	10
	Synthetic data	25
